# A Narrative Review on the Impact of Smoking on Female Fertility

**DOI:** 10.7759/cureus.58389

**Published:** 2024-04-16

**Authors:** Vaishnavi D Dhage, Nikhilesh Nagtode, Dimple Kumar, Arpana K Bhagat

**Affiliations:** 1 Department of Community Medicine, Jawaharlal Nehru Medical College, School of Epidemiology and Public Health, Datta Meghe Institute of Higher Education and Research, Wardha, IND

**Keywords:** implantation, ovary, reproductive function, female infertility, female fertility, cigarette smoking, smoking

## Abstract

Understanding the significant impact of preventable factors, such as lifestyle decisions and bad habits like smoking, on female fertility has received a lot of attention. Pervasive smoking among fertile women is a serious public health concern. Smoking has well-documented negative impacts on general health, but it also has significant consequences on fertility. Many women of reproductive age still smoke, despite a wealth of data elucidating the effects of pregnancy and the health of the fetus as a result of prenatal exposure. This review attempts to investigate the consequences of smoking on female fertility, specifically focusing on how it affects the ovaries, oviducts, and uterus through a thorough examination of numerous studies. Important topics such as ovarian reserve, steroidogenesis, ovulation, controlling the menstrual cycle, oviductal function, uterine receptivity, and implantation will receive extra focus.

## Introduction and background

International literature has documented a notable trend over the past 50 years facing an advanced global loss in human fertility; consequently, a considerable amount of focus has been placed on identifying risk factors that can be modified by lifestyle and environment that impact human reproductive function. At the moment, a comprehensively recognized definition of infertility is the failure to attain a clinical pregnancy after one year of trying as well as unprotected sexual activity [1,2]. Approximately 37% of infertility cases are attributed to female factors, while 29% are attributed to male factors. The remaining 18% of cases comprehend a combination of both male and female factors. Genetic factors account for 1% of the causes, leaving the remaining 16% to be explained by idiopathic infertility, which is diagnosed when there is no clear cause. Infertility in women can be caused by ovarian, oviductal, and uterine problems [3,4].

It has long been established that leading an unhealthy lifestyle harms one's health. Tobacco use is the chief preventable risk factor for several diseases that affect people, and it also increases mortality as well as morbidity [1,3]. The smoking prevalence among adult women is 15.3% overall, with 20.7% of women smoking between the ages of 25 and 44 [4]. Cigarette smoking among women has become more prevalent globally in recent times, raising concerns about public health [5]. The findings indicate that friends and family who smoke, as well as joining the family in smoking, are factors contributing to the rise in smoking rates among both men and women [6]. While most existing data surrounding the effects of smoking on female fertility comes from past studies (retrospective), recent years have witnessed a growing concern and exploration of this topic. This emerging research suggests a significant alliance between smoking, both active and passive, and lowered fertility in women. Furthermore, evidence indicates a potential link between prenatal exposure to smoke and an augmented risk of adverse pregnancy outcomes and decreased fertility in the female offspring later in life [1]. These data suggest that pregnant and fertility-disordered women should be sent to smoking cessation programs or given advice on quitting smoking [7]. This review discusses the consequences of smoking on female fertility.

## Review

Methodology

This review primarily focuses on the impact of cigarette smoking on female fertility. Articles were sourced through literature searches on Google Scholar, PubMed (MEDLINE), and WHO websites in January 2024, using terms like "smoke," "smoking," "smoker," and "cigarette," combined with terms related to female reproductive health such as "female fertility," "female infertility," "reproductive function," "ovary," and "implantation." Additionally, relevant references such as experimental, nonexperimental, and literature reviews were identified by examining citations in pertinent studies. Only previous studies published in English before January 2024 were considered, with those in other languages being excluded. Various filters, including full text and free full text, were utilized during the search process.

Discussion

Smoking and Female Fertility

It has been repeatedly shown that smoking contributes to a broad spectrum of human health issues, encompassing reproductive irregularities, making it a global health concern [7-9]. Despite the compelling evidence regarding smoking's harmful effects on female fertility and the impressive antismoking initiatives spearheaded by healthcare providers, 175 million women globally who are 15 years of age or older currently smoke, either daily or occasionally [4,10]. Around 4000 different chemical classes, including polycyclic aromatic hydrocarbons, heavy metals, and alkaloids, all exhibiting reproductive toxicity, are found in cigarette smoke. As a result, cigarette smoke comprises a complex blend of substances that may have combined impacts on different components of the reproductive system [11-14]. An extensive review of research has found a strong link between smoking and reduced fertility. This suggests that smokers may be up to 60% more likely to encounter infertility analogized to nonsmokers [15]. Strong evidence indicates that smoking has a variety of distinct effects on the ovary, oviduct, and uterus, among other targets, affecting numerous components of the female reproductive system [11,16,17]. The consequences of smoking on the reproductive function in women are depicted in Table 1 [1].

**Table 1:** Consequences of smoking on the reproductive function in women

Sr. no.	Components of the female reproductive system	Consequences of smoking on the reproductive function in women
1.	Ovary	Early menopause, poor quality oocytes
2.	Steroidogenesis	Decreased estrogen and progesterone levels, increased androgens
3.	Oviduct	Increased ectopic pregnancy, affected oviductal smooth muscles
4.	Uterus/implantation	Delayed implantation, decreased uterus receptivity
5.	Menstrual cycle	Increased oligomenorrhea, increased dysmenorrhea, heavy bleeding

Smoking and Ovarian Reserve

The phrase "ovarian reserve" refers to the ovary's ability to produce egg cells that can be fertilized and result in a successful and healthy pregnancy [18]. Research has shown that women's ovarian reserve can decrease by about 20% as a result of smoking cigarettes [19]. The correlation between smoking and the inability to conceive could be partially explained by all of the previously mentioned correlations between smoking and ovarian or hormonal abnormalities. One factor that unites the previously listed anomalies is polycystic ovary syndrome, which has been linked to smoking [20]. The cross-sectional study conducted in North Carolina (2010) is the first study to mainly ascertain how smoking exposure affects levels of anti-Müllerian hormone (AMH). Reduced levels of AMH were linked to current smoking, indicating a direct correlation between smoking and ovarian follicular depletion [21]. According to the findings, women who smoke have a negative correlation with their ovarian reserve, as determined by their follicular phase blood follicle-stimulating hormone concentrations and how they react to ovarian stimulation during in-vitro fertilization treatment [22].

Smoking and Steroidogenesis

Certain chemicals found in cigarette smoke have the characteristics of endocrine disruptors. Smokers exhibit an aberrant endocrine profile after ovarian stimulation in in-vitro fertilization, which is typified by increased testosterone and decreased estradiol levels. Numerous authors have noted that smoke-related substances interfere with steroidogenesis, impairing the production of progesterone and estrogen. Cigarette smoke contains chemicals that can alter hormones in a variety of ways. In addition, some compounds have been shown to have the opposite impact, such as estrogenic and antiestrogenic effects. Actions on the distinct phases and targets of steroidogenesis may account for these opposing effects. To fully understand the effects of cigarette smoke on steroidogenesis, research into the intricacy of the smoke component's impacts is necessary [11].

Smoking and Ovulation

The biological process known as ovulation occurs when the ovary releases a mature ovum, which travels to the fallopian tube and remains there until the sperm fertilizes it. Ovulation is harmed by smoking. The harmful component in cigarettes lowers the levels of progesterone and estrogens, the female hormones. Consequently, there are fewer mature eggs available for fertilization. It can also destroy and decrease the quantity of eggs in the ovaries. Smoking can lower the quality of eggs and induce irregular growth after fertilization, even if it doesn't kill the eggs. Additionally, smoking can harm the genetic material in eggs, raising the possibility of miscarriages and birth abnormalities [23].

Smoking and Menstruation

Smoking likely causes oligomenorrhea. The data suggests that the strongest correlation between smoking and oligomenorrhea occurs in women between the ages of 40 and 49. This finding follows other data showing how smoking damages the ovaries [20]. The findings of the meta-analysis conducted in China (2020) provide compelling evidence that smoking cigarettes is substantially associated with a higher incidence of dysmenorrhea in fertile women [24].

Smoking and Oviduct Function

The oviduct, a tubelike structure that connects the ovary to the uterus, is a component of the female reproductive tract [25]. According to the findings of the meta-analysis conducted in China (2020), smoking before and throughout the first trimester of pregnancy slows down the flow of embryos via the oviduct [24]. According to the findings of the retrospectively analyzed study conducted in Spain (2006), smokers have also been found to have a higher incidence of ectopic pregnancies, and research using animal models has demonstrated that tobacco use affects tubal or oviductal function [26].

Smoking and Uterus Receptivity

According to the findings of the retrospectively analyzed study conducted in Spain (2006), smoking cigarettes has a detrimental impact on uterine receptiveness apart from its influence on ovarian function, as seen by the plenty of reduced conception rate observed in heavy smokers. These results imply that various modifications are made to uterine function, either mechanically or biochemically. To determine the exact effect of smoking on uterine receptivity, a retrospective study that ruled out confounding variables such as smoking's effects on the ovary, oocyte quantity, and quality, embryo quality, as well as oviductal transit was conducted [26].

Smoking and Implantation

Implantation is the process by which the embryo sticks to the endometrial surface of the uterus, invades the epithelium, and finally enters the mother's circulation to develop into the placenta [27]. The findings of original study conducted in the USA (2011) showed that exposure to a mother who is smoking during pregnancy was linked to earlier implantation, and current smoking was linked to later implantation. Smokers may experience delayed implantation as a result of tubal transfer or oviductal interruption of ovum retrieval. According to research on in-vitro fertilization, smoking by mothers may also negatively impact several other reproductive processes. Smoking has been linked to lower rates of implantation, fertilization, and ova retrieval as well as an increased chance of miscarriage [28].

Smoking and Conception Delay

According to study published in the USA (2018), there was a positive correlation between the number of cigarettes smoked daily and the delay in pregnancy. Compared to nonsmokers, smokers had a 54% greater risk of women reporting conception delays lasting longer than 12 months. The consequences of passive cigarette smoke exposure alone were only marginally less severe than those of active smoking by either partner, or both partners' active smoking had negative effects. The majority of research backs up the conclusion that smokers have greater rates of infertility, lower fecundity, and longer time to conception than nonsmokers [4].

Smoking and Assisted Reproductive Technologies (ART)

Several observational studies assessed how smoking affected the results of ART or natural female fertility; most of the research confirmed the evidence of a higher incidence of infertility among smokers. It has been discovered that passive smoking has the same unfavorable effects on implantation and pregnancy rates as active smoking [29]. The meta-analysis conducted in the United Kingdom (2008) offers proof that smoking cigarettes has a markedly detrimental impact on the clinical results of ART. In addition to the clear indication of a detrimental impact on the rates of live births, miscarriages, ectopic pregnancies, and fertilization, there is particularly compelling evidence for a lower clinical pregnancy rate among smokers. Women who are currently smoking and seeking infertility treatment should be shown this research to increase the success rates of ART. They should also be strongly advised to stop smoking [30].

Smoking and Fecundity

The term "fecundity" describes the capacity to become pregnant and bring a child to term, whereas "fecundability" quantifies the likelihood of being pregnant each menstrual cycle. For a long time, smoking has been the most likely component cause of fecundity. Small dosages of nicotine administered to female rats cause decreased fertility and smaller-than-expected litter sizes. The first evidence of a smoking influence on fecundity in human populations dates back to 1983. Smoking was found to lower fecundity in couples whose infertility had no discernible medical cause [31].

Smoking and Miscarriage

While some evaluations of the literature that have been published since the 1970s have found that smoking causes miscarriage, the majority have highlighted the inconsistent data about smoking's link to miscarriage. The relationship between a mother smoking during pregnancy and the risk of miscarriage was validated by the systematic review and meta-analysis conducted in California (2014), which also demonstrated that the risk rises with smoking frequency. The findings support the hypothesis that exposure to secondhand smoke has either no effect or a slight influence on the chance of miscarriage [32].

Smoking and Menopause

One lifestyle factor that may impact the timing of menopause is smoking. According to a systematic review and meta-analysis conducted in Australia (2014), smokers had a 33% higher chance than nonsmokers of going through the postmenopausal stage at a given age. From a biological perspective, smoking has been linked to the synthesis and metabolism of hormones, including gene expression, lowered serum estrogen levels, elevated 2-hydroxyestrogen concentrations, and increased androgen quantity, all of which have an antiestrogen effect that causes earlier natural menopause. The results support the conclusions from other research that an earlier age at natural menopause is associated with current smoking [33]. Women who smoke experience menopause 1-4 years earlier than women who do not smoke [4].

Effects of Smoking During Pregnancy on Infants

Additionally, studies have indicated that pregnant women who smoke increase the risk of issues in infants, such as premature delivery, low-birth-weight (LBW) babies, stillbirth, and infant fatalities [34].

Recommendations

Healthcare professionals and public health professionals can support smoking cessation by offering education, continuous monitoring, and personalized support to individuals striving to quit. Of all the groups in society, health professionals and public health professionals have the most ability to encourage a decrease in tobacco usage such as smoking. Professionals in the field can boost the likelihood of successfully quitting by up to 30% with brief advice and 84% with thorough advice. 

## Conclusions

While there is thriving interest in understanding how avoidable lifestyle aspects affect female reproductive function, the impact of smoking remains a contentious issue, with studies yielding conflicting results and inherent limitations preventing definitive causal conclusions. Exposure to cigarette smoke detrimentally affects various stages of the reproductive process and components of the reproductive system, including ovarian reserve, steroidogenesis, ovulation, menstruation, oviduct function, uterine receptivity, implantation, as well as early placentation. The compounds in cigarette smoke interact with different aspects of the reproductive system, influenced by individual sensitivities, concurrent exposure to other toxins, and factors such as timing, dosage, type, and length of susceptibility. Given that women experiencing infertility are often motivated to quit smoking, healthcare providers should actively encourage cessation efforts in these patients.

## References

[1] de Angelis C, Nardone A, Garifalos F (2020). Smoke, alcohol and drug addiction and female fertility. Reprod Biol Endocrinol.

[2] Zegers-Hochschild F, Adamson GD, Dyer S (2017). The International Glossary on Infertility and Fertility Care, 2017. Hum Reprod.

[3] (2024). State-Specific Smoking-Attributable Mortality and Years of Potential Life Lost --- United States, 2000--2004. https://www.cdc.gov/mmwr/preview/mmwrhtml/mm5802a2.htm.

[4] Practice Committee of the American Society for Reproductive Medicine (2018). Smoking and infertility: a committee opinion. Fertil Steril.

[5] Jafari A, Rajabi A, Gholian-Aval M, Peyman N, Mahdizadeh M, Tehrani H (2021). National, regional, and global prevalence of cigarette smoking among women/females in the general population: a systematic review and meta-analysis. Environ Health Prev Med.

[6] Johnston V, Westphal DW, Earnshaw C, Thomas DP (2012). Starting to smoke: a qualitative study of the experiences of Australian indigenous youth. BMC Public Health.

[7] National Collaborating Centre for Women’s and Children’s Health (UK) (2024). Fertility: assessment and treatment for people with fertility problems. Fertility: Assessment and Treatment for People with Fertility Problems.

[8] (2024). Health Effects of Cigarette Smoking. https://www.cdc.gov/tobacco/data_statistics/fact_sheets/health_effects/effects_cig_smoking/index.htm.

[9] Sansone A, Di Dato C, de Angelis C (2018). Smoke, alcohol and drug addiction and male fertility. Reprod Biol Endocrinol.

[10] (2024). Tobacco. https://www.who.int/news-room/fact-sheets/detail/tobacco.

[11] Dechanet C, Anahory T, Mathieu Daude JC (2011). Effects of cigarette smoking on reproduction. Hum Reprod Update.

[12] de Angelis C, Galdiero M, Pivonello C (2017). The environment and male reproduction: the effect of cadmium exposure on reproductive function and its implication in fertility. Reprod Toxicol.

[13] Rzymski P, Tomczyk K, Rzymski P, Poniedziałek B, Opala T, Wilczak M (2015). Impact of heavy metals on the female reproductive system. Ann Agric Environ Med.

[14] Alviggi C, Guadagni R, Conforti A (2014). Association between intrafollicular concentration of benzene and outcome of controlled ovarian stimulation in IVF/ICSI cycles: a pilot study. J Ovarian Res.

[15] Augood C, Duckitt K, Templeton AA (1998). Smoking and female infertility: a systematic review and meta-analysis. Hum Reprod.

[16] Budani MC, Tiboni GM (2017). Ovotoxicity of cigarette smoke: a systematic review of the literature. Reprod Toxicol.

[17] Talbot P, Riveles K (2005). Smoking and reproduction: the oviduct as a target of cigarette smoke. Reprod Biol Endocrinol.

[18] (2024). Ovarian reserve. https://en.wikipedia.org/wiki/Ovarian_reserve.

[19] Cui J, Wang Y (2024). Premature ovarian insufficiency: a review on the role of tobacco smoke, its clinical harm, and treatment. J Ovarian Res.

[20] Hartz AJ, Kelber S, Borkowf H, Wild R, Gillis BL, Rimm AA (1987). The association of smoking with clinical indicators of altered sex steroids--a study of 50,145 women. Public Health Rep.

[21] Plante BJ, Cooper GS, Baird DD, Steiner AZ (2010). The impact of smoking on antimüllerian hormone levels in women aged 38 to 50 years. Menopause.

[22] El-Nemr A, Al-Shawaf T, Sabatini L, Wilson C, Lower AM, Grudzinskas JG (1998). Effect of smoking on ovarian reserve and ovarian stimulation in in-vitro fertilization and embryo transfer. Hum Reprod.

[23] (2024). How does smoking impact ovulation? expert explains. https://www.herzindagi.com/advice/impact-of-smoking-on-ovulation-by-expert-article-199419.

[24] Qin LL, Hu Z, Kaminga AC, Luo BA, Xu HL, Feng XL, Liu JH (2020). Association between cigarette smoking and the risk of dysmenorrhea: a meta-analysis of observational studies. PLoS One.

[25] Li S, Winuthayanon W (2017). Oviduct: roles in fertilization and early embryo development. J Endocrinol.

[26] Soares SR, Simon C, Remohí J, Pellicer A (2007). Cigarette smoking affects uterine receptiveness. Hum Reprod.

[27] Kim SM, Kim JS (2017). A review of mechanisms of implantation. Dev Reprod.

[28] Jukic AM, Weinberg CR, Baird DD, Wilcox AJ (2011). The association of maternal factors with delayed implantation and the initial rise of urinary human chorionic gonadotrophin. Hum Reprod.

[29] Neal MS, Hughes EG, Holloway AC, Foster WG (2005). Sidestream smoking is equally as damaging as mainstream smoking on IVF outcomes. Hum Reprod.

[30] Waylen AL, Metwally M, Jones GL, Wilkinson AJ, Ledger WL (2009). Effects of cigarette smoking upon clinical outcomes of assisted reproduction: a meta-analysis. Hum Reprod Update.

[31] Bolumar F, Olsen J, Boldsen J (1996). Smoking reduces fecundity: a European multicenter study on infertility and subfecundity. Am J Epidemiol.

[32] Pineles BL, Park E, Samet JM (2014). Systematic review and meta-analysis of miscarriage and maternal exposure to tobacco smoke during pregnancy. Am J Epidemiol.

[33] Schoenaker DA, Jackson CA, Rowlands JV, Mishra GD (2014). Socioeconomic position, lifestyle factors and age at natural menopause: a systematic review and meta-analyses of studies across six continents. Int J Epidemiol.

[34] Office of the Surgeon General (US), Office on Smoking and Health (US) (2024). The health consequences of smoking. The Health Consequences of Smoking: A Report of the Surgeon General.

